# A new evaluation system for drug–microbiota interactions

**DOI:** 10.1002/imt2.199

**Published:** 2024-05-07

**Authors:** Tian‐Hao Liu, Chen‐Yang Zhang, Hang Zhang, Jing Jin, Xue Li, Shi‐Qiang Liang, Yu‐Zheng Xue, Feng‐Lai Yuan, Ya‐Hong Zhou, Xiu‐Wu Bian, Hong Wei

**Affiliations:** ^1^ Yu‐Yue Pathology Scientific Research Center Chongqing China; ^2^ Department of Pathology Army Medical University Chongqing China; ^3^ Department of Gastroenterology Affiliated Hospital of Jiangnan University Wuxi Jiangsu China; ^4^ Institute of Integrated traditional Chinese and Western Medicine Affiliated Hospital of Jiangnan University Wuxi China; ^5^ College of Animal Science and Technology, College of Animal Medicine Huazhong Agricultural University Wuhan Hubei China; ^6^ Wuxi Hospital Affiliated to Nanjing University of Chinese Medicine Wuxi Jiangsu China

**Keywords:** drug metabolism, drug–microbiota interactions, genetic and environmental factors, gut microbiota

## Abstract

The drug response phenotype is determined by a combination of genetic and environmental factors. The high clinical conversion failure rate of gene‐targeted drugs might be attributed to the lack of emphasis on environmental factors and the inherent individual variability in drug response (IVDR). Current evidence suggests that environmental variables, rather than the disease itself, are the primary determinants of both gut microbiota composition and drug metabolism. Additionally, individual differences in gut microbiota create a unique metabolic environment that influences the in vivo processes underlying drug absorption, distribution, metabolism, and excretion (ADME). Here, we discuss how gut microbiota, shaped by both genetic and environmental factors, affects the host's ADME microenvironment within a new evaluation system for drug–microbiota interactions. Furthermore, we propose a new top‐down research approach to investigate the intricate nature of drug–microbiota interactions in vivo. This approach utilizes germ‐free animal models, providing foundation for the development of a new evaluation system for drug–microbiota interactions.

## Highlights

- Shaped by both genetic and environmental factors, gut microbiota significantly influences the host's drug absorption, distribution, metabolism, and excretion (ADME) microenvironment, highlighting the need for a new evaluation system for drug‐microbiome interactions.
- To investigate the intricate nature of interactions between gut microbiota and drugs in vivo, a novel top‐down research approach utilizing germ‐free animal models is proposed.

## Graphical abstract

Here, we propose a new evaluation system and research strategy for drug microbial interaction. Leveraging precision germ‐free animal models for the design of next‐generation drug active/toxic ingredients involving gut microbiota, as well as microbes sorting and cultromics, to elucidate the exact mechanism of interaction between single microbe and single drug ingredient in vivo.

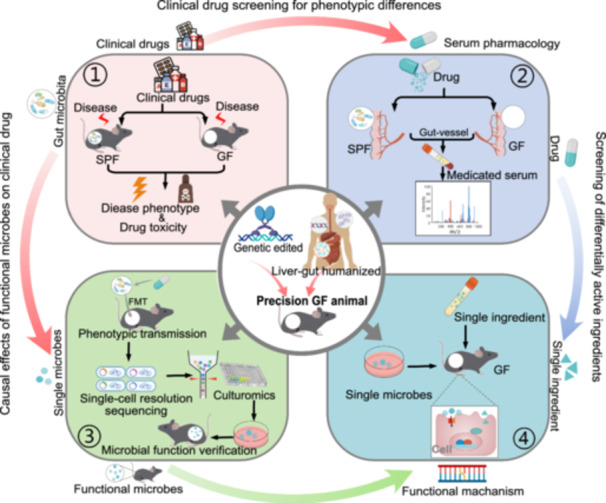

## INTRODUCTION

**Figure 1:**
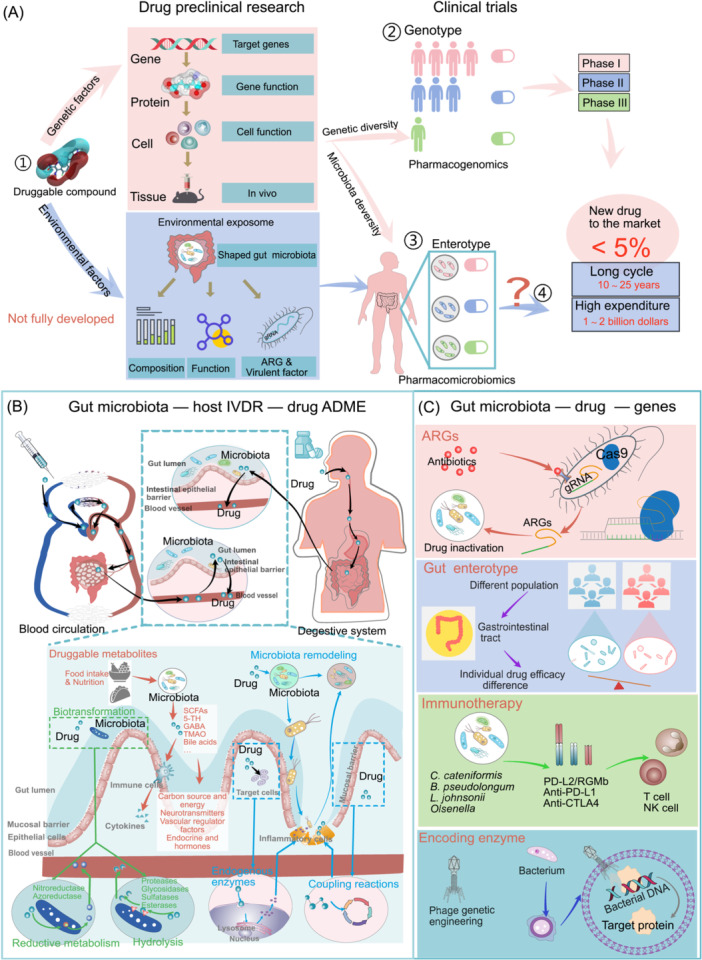
The clinical significance of gut microbiota in pharmacological research and the regulation of drug‐targeting genes. (A) *Pharmacomicrobiomics* provides new opportunities for preclinical investigations of drug efficacy and safety. ① The effect of drugs on disease characterization depends on a combination of genetic and environmental factors. ② *Pharmacogenomics* employs human genomic information (genetic factors) to decode the reasons behind individual variability in drug response (IVDR). ③ *Pharmacomicrobiomics* employs gut microbial genomic information (environmental factors) to decode the reasons behind environmental factors in drug response. ④ A better understanding of the role of environmental factors on drug safety and efficacy is expected to provide preclinical research strategies to improve the success of clinical drug development. (B) Involvement of gut microbiota in drug ADME. Up: Gut microbiota is a primary site for the drug ADME. Whether the drug is administered orally or injected, it directly reaches the gut microbiota or via the bloodstream. Down: Gut microbiota produced new drugs or druggable metabolites. It concludes short‐chain fatty acids (SCFAs), vitamins, 5‐hydroxytryptamine (5‐TH), γ‐aminobutyric acid (GABA), trimethylamine oxide (TMAO), and bile acids, as well as secondary metabolites of related metabolic pathways. Gut microbiota is involved in a variety of metabolic reactions, mainly involving reductive metabolism and hydrolysis. Drug‐induced gut microbiota remodeling. Most drugs are metabolized through endogenous enzymes and coupling reactions, resulting in ectopic microbiota colonization and remodeling of the gut microbiota. (C) Interactions between gut microbiota genes and drug‐targeted host genes. ARGs: Drugs can influence the expression of antibiotic resistance genes (ARGs) in the gut microbiota. Gut phenotype: The relationship between personalized gut phenotype and drug efficacy based on genetic sequencing of the gut microbiota has been demonstrated. Immunotherapy: Gut microbiota can modulate the safety and efficacy of targeted immunomodulators. Encoding enzyme: CRISPR gene editing of engineered bacteria targets the elimination of ARGs in gut microbiota.

Since the introduction of pharmacogenetics by Dr. *Archibald Edward Garrod* in the early 20th century, the influence of human genetic information on drug response (individual variability in drug response [IVDR]) has been increasingly emphasized. *Pharmacogenomics* harnesses this information to understand variations in drug sensitivity, metabolism, and adverse drug reactions (ADRs) [1]. In recent years, clinical drug research has primarily focused on the development of new drugs targeting disease gene products. Despite the emergence of numerous new drugs, translating innovative ideas into effective and safe therapeutics remains a challenge. Over 50% of Phase III trials have failed to replicate positive results from Phase II, leading to wasted resources and potential harm to participants [2]. It is now understood that the drug response phenotype is determined by a complex interplay of genetic and environmental factors. Gut microbiota composition reflects environmental influences such as diet, lifestyle, medication use, and calorie intake [3, 4]. This study aims to highlight recent advancements in pharmacology regarding how gut microbiota, shaped by both genetic and environmental factors, affects the host's drug absorption, distribution, metabolism, and excretion (ADME) microenvironment within a new framework for evaluating drug–microbiota interactions. By incorporating gut microbiota, we may gain new opportunities for preclinical drug efficacy and safety investigations (Figure 1A).

*Pharmacogenomics* leverages human genomic information to decode the mechanisms underlying IVDR, particularly focusing on the in vivo processes of drug ADME. Similarly, *Pharmacomicrobiomics* aims to understand how gut microbiota genomic information contributes to IVDR. Current research has predominantly focused on identifying drug‐metabolizing enzymes produced by gut bacteria through isolation [5, 6]. However, the metabolic function of gut microbiota is influenced by the host microenvironment, as the gut microbiota itself is a key component of this complex environment. This intricate interplay between gut microbiota and the host environment may be the key reason for its involvement in IVDR. This study innovatively investigates how gut microbiota alters the host microenvironment's impact on ADME, focusing on the in vivo function of gut microbiota within the host body. Additionally, the study proposes a new top‐down research approach to address the challenges of *Pharmacomicrobiomics*. Overall, *Pharmacomicrobiomics* offers not only new insights into the mechanisms of IVDR but also new standards for preclinical drug research. By considering both genetic and environmental factors (as reflected by gut microbiota), this field holds promise. Furthermore, due to the relative ease of manipulating gut microbiota and its high safety profile, *Pharmacomicrobiomics* has the potential to provide effective solutions for improving drug efficacy and reducing toxicity through the development of a new evaluation system for drug–microbiota interactions.

### How does gut microbiota drive individual differences in drug ADME?

Drugs circulating and undergoing metabolism in the bloodstream can reside in the gut for extended periods, where crucial drug–microbiota interactions occur. This interaction is bidirectional: drugs can alter gut microbial composition, growth, and function, while gut microbiota can metabolize and modify drug structure through enzymatic activity. These interactions ultimately influence a drug's bioavailability, biological activity/toxicity, and consequently, the individual's response to the medication. Regardless of the administration route (oral or injected), drugs can directly reach the gut microbiota or indirectly interact with it via the bloodstream. Although studies have identified drug‐metabolizing enzymes derived from gut microbiota [5, 6], individual responses to interventions like fecal microbiota transplantation (FMT) and probiotics vary significantly [7]. ADME processes determine a drug's stability, target site delivery, potential for generating toxic substances, and successful elimination of excess components. Individual variations in the gut microbiota‐influenced metabolic environment in vivo can influence drug ADME, regardless of the administration route (Figure 1B).

### Gut microbiota produces druggable metabolites

The gut microbiota exerts a broad and profound influence on the physiological functions of various bodily systems. Individual differences in gut microbiota composition create a unique metabolic environment within the body, impacting drug ADME. Furthermore, the secondary metabolites produced by gut microbiota can serve as druggable targets for regulating bodily functions. Through fermentation, gut microbiota digest complex carbohydrates and proteins, generating vitamins and enteral nutrients. These nutrients modulate host energy metabolism and serve as a carbon source for colonocytes [8]. A clinical medication study even demonstrated a clear association between polypharmacy and microbial functions related to short‐chain fatty acid (SCFA) metabolism [9]. The dynamic interplay between gut microbiota and bile acid biotransformation plays a key role in maintaining glycolipid metabolic homeostasis, while also modulating intestinal immunity, inflammation, and tumorigenesis [10, 11]. These microbiota‐derived metabolites also contribute to the maintenance of liver and kidney function, which are crucial organs for drug metabolism and excretion.

### Gut microbiota biotransformation of drugs

The gut microbiota plays a crucial role in various metabolic reactions through enzymes. These reactions include reduction, hydrolysis, functional group transfer, and cleavage. These complex metabolic processes can regulate the duration and intensity of a drug's pharmacological effects, ultimately influencing its clinical benefit [6]. Studies have shown that, beyond antibiotics, many nonantibiotic drugs targeting humans can also be affected by gut microbiome metabolism. This includes medications like statins and digoxin [12]. Gut microbiota can directly degrade or compete with drugs by producing drug‐degrading enzymes [13, 14]. Indirectly, drugs can influence the activity of gut microbiota's drug‐metabolizing enzymes and metabolic pathways, either activating or inactivating them through host drug‐metabolizing enzymes [15, 16]. This concept even applies to reducing drug side effects. For example, higher statin doses increase the risk of liver and muscle problems, but combining them with probiotics can potentially lower the effective dose and minimize these side effects [17].

### Drug‐induced gut microbiota remodeling

Most drugs undergo metabolism through endogenous enzymes and conjugation reactions. This process can have unintended consequences, leading to changes in gut microbiota composition and colonization patterns. Drugs can disrupt the mucosal barrier of the gut, oral cavity, and gastrointestinal tract in two ways. On the one hand, they can trigger inflammation and promote the ectopic colonization of microbiota in unintended locations [18, 19]. For instance, methotrexate, a common immunosuppressive and anticancer drug, can significantly reduce levels of *Bacteroides fragilis* while increasing macrophage density in the gut, leading to gastrointestinal harm in clinical settings [20]. Conversely, some drugs can have beneficial effects on the gastrointestinal mucosal barrier. Metformin, for example, promotes the production of short‐chain fatty acids by gut microbiota, which strengthens intestinal mucosal immunity and contributes to its therapeutic effects in reducing insulin resistance and promoting blood glucose homeostasis [21]. These examples highlight how gut microbiota can influence individual differences in drug absorption, distribution, metabolism, and excretion. However, the precise mechanisms by which drugs contribute to these individual variations remain under investigation.

### How do drug‐targeted host genes contribute to the gut microbiota?

*Pharmacomicrobiomics* has emerged as a natural extension of *pharmacogenomics*, driven by the growing recognition of gut microbiota as a “second human genome” (Figure 1C). A major concern associated with antibiotic use is the widespread presence of antibiotic resistance genes (ARGs) within gut microbiota. The human gut serves as a reservoir for resistance to β‐lactam and plasmid‐mediated quinolone antibiotics [22]. It has been established that antibiotic use can enrich the abundance of resistant bacteria and ARGs within the gut microbiota. Interestingly, similar effects have been observed with nonantibiotic drugs, including nonsteroidal anti‐inflammatory drugs (NSAIDs) such as ibuprofen, naproxen, and diclofenac, as well as the antiepileptic drug carbamazepine [23, 24]. Clinical trials are exploring the use of gene‐edited bacteria, called clustered regularly interspaced short palindromic repeats (CRISPR) systems, to eliminate antibiotic‐resistant bacteria. Current evidence suggests that gut microbiota facilitates the transmission of antibiotic resistance plasmids within the human gut [25]. Conversely, novel derivatives obtained by manipulating gut microbiota using techniques like immobilization, enzyme‐directed evolution, flow biocatalysis, protein engineering, and combinatorial biosynthesis hold promise in combating the spread of antibiotic resistance [26]. For example, FMT and colonization with the gut bacterium *Coprobacillus cateniformis* have been shown to improve the efficacy of PD‐1 immunotherapy by downregulating the PD‐L2‐RGMb pathway [27]. Recent studies have confirmed the association between specific gut microbiota enterotypes and drug efficacy [28]. Characterizing an individual's gut microbiota enterotype has the potential to inform personalized and precision medicine approaches. While it has been established that drug‐targeted host genes influence gut microbiota, the specific environmental factors involved require further investigation.

### Influence of environmental variables on gut microbiota and its implications for developing a novel drug–microbiota interaction evaluation system

**Figure 2:**
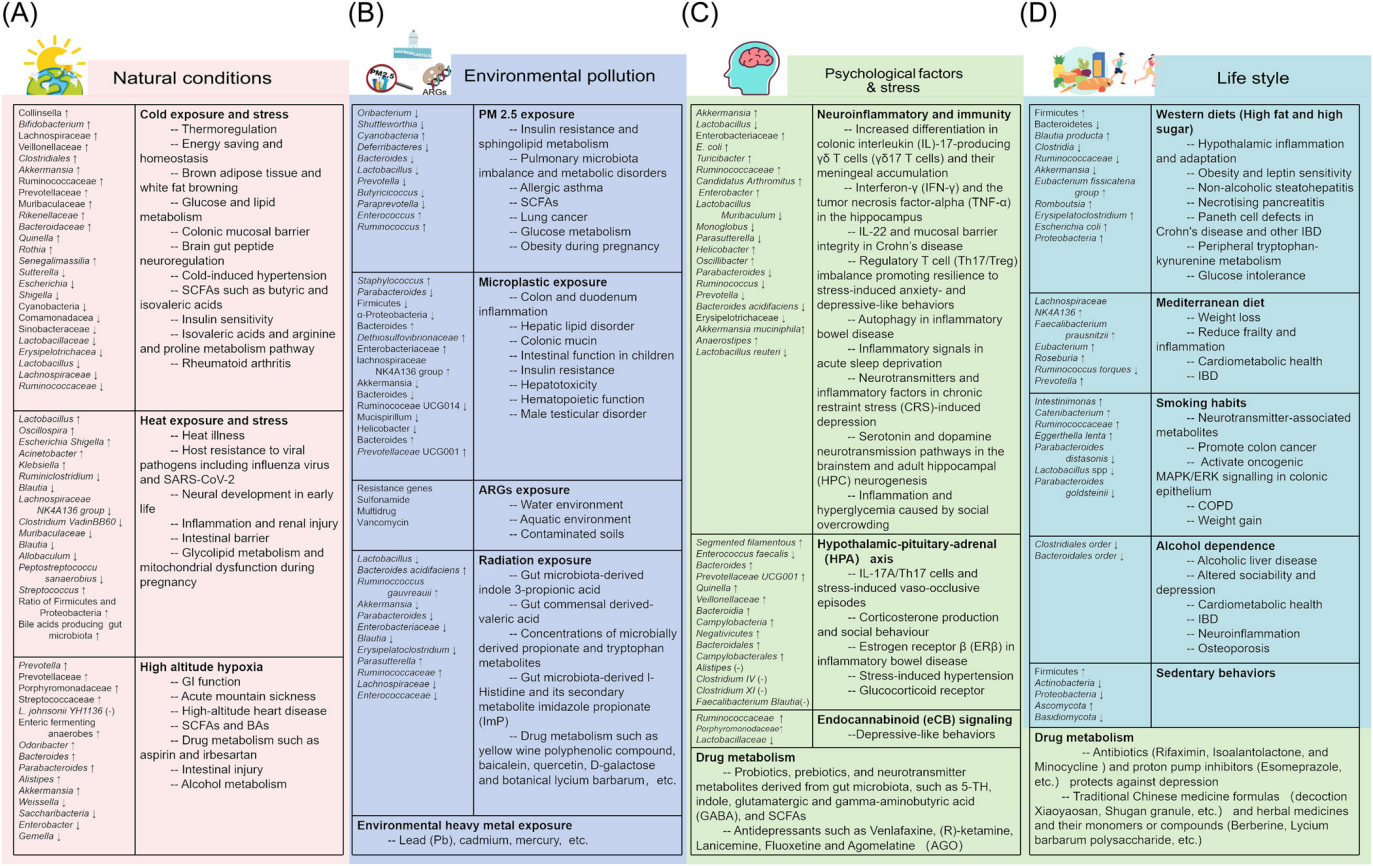
Environmental factors influencing gut microbiota. (A) Natural conditions: natural environment contributions to the gut microbiota. The climatic extremes including cold and heat exposure or stress and high‐altitude hypoxia environment can alter the specific bacteria in gut and have an impact on the host response to extreme environments. (B) Environmental pollution: Environmental contamination including PM2.5 pollution, microplastic pollution, and antibiotic resistance genes (ARGs) contributes to the gut microbiota and human health. (C) Psychological factor and stress: There is an important relationship between gut microbiota and cognitive and behavioral processes caused by psychological stress, which mainly include hypothalamic‐pituitary‐adrenal axis (HPA‐axis), neuroimmune regulation, immune system, and endocannabinoid (eCB). Moreover, psychiatric factors are one of the most important influences on drug metabolism. (D) Lifestyle: The intervention effect of lifestyle including western diets, modern lifestyles, sedentary behaviors, smoking habits, and alcohol dependence on the gut microbiota is evident.

While correcting environmental factors is crucial, investigating drugs that can mitigate their negative effects on the gut microbiota is equally important. Mounting evidence in recent years has linked gut microbial composition and function to a wide range of diseases. For instance, a 2022 cohort study published in *Nature* involving 8208 participants found that various environmental factors, both during early life and present times, significantly affect gut microbial composition and function. In fact, the study suggests that environmental factors may have an even greater impact on the gut microbiota than the disease itself [4]. These environmental factors encompass both the external natural environment and social environment (Figure 2).

### Natural environment contributions to the drug–microbiota interactions

Natural climate and geographic environments shape unique microbial ecosystems, facilitating the direct transfer of microbes to humans. Furthermore, climatic extremes can influence the diversity of gut microbiota composition. Interestingly, specific gut microbes can undergo adaptive changes that benefit the host's response to these extreme environments. For example, factors like low temperature, high heat, and high altitude can all influence the composition of gut microbiota, leading to changes in the activity of drug‐metabolizing enzymes harbored by these gut microbes [29, 30, 31]. Additionally, hypoxia can impact the direct and indirect effects of gut microbiota on its own or host drug metabolism. These effects are mediated by various factors, including gut microbiota‐derived drug‐metabolizing enzymes, metabolites, and immune modulation [32].

### Environmental contamination contributions to the drug–microbiota interactions

External environmental contaminants, including ARGs, PM2.5 particulates, and microplastics, may represent another significant factor influencing drug metabolism within the gut microbiota. It is important to note that ARGs and pollution do not directly inactivate antibiotic efficacy, but rather contribute to the development and spread of antibiotic‐resistant bacteria, thereby reducing the effectiveness of these drugs [33, 34, 35]. The metabolic function of gut microbiota is a critical factor influencing both drug effectiveness and the potential for side effects. For instance, studies have shown that ephedra polysaccharides can alleviate PM2.5‐induced airway inflammation resembling asthma in mice by modulating gut microbiota composition and SCFA production [36]. Microplastics can disrupt gut microbiota metabolism, potentially contributing to host insulin resistance, liver damage, and blood cell abnormalities [37, 38, 39]. Interestingly, gut microbiota also plays a role in metabolizing and influencing the efficacy of drugs used to mitigate radiation damage. Examples include yellow wine polyphenolic compounds, baicalein, quercetin, d‐galactose, and the botanical *Lycium barbarum* [40, 41, 42, 43].

### Social environmental factors contribute to the drug–microbiota interactions

The gut microbiota plays a crucial role in cognitive and behavioral processes influenced by psychological stress. This interplay primarily involves the hypothalamic‐pituitary‐adrenal axis (HPA axis), neuroimmune regulation, and the immune system. Compelling evidence suggests that gut commensal segmented filamentous bacteria can exacerbate cardiovascular disorders associated with psychological stress by influencing the HPA axis [44, 45]. Research also indicates that chronic stress can alter the expression and activity of drug‐metabolizing enzyme genes within gut microbiota [46]. Gut microbiota‐derived metabolites, including probiotics, prebiotics, and neurotransmitters such as 5‐hydroxytryptophan (5‐HT), indole, glutamate, gamma‐aminobutyric acid (GABA), and SCFAs, can alleviate chronic stress‐induced cognitive impairment and emotional responses [47, 48]. Interestingly, the efficacy of clinical antidepressants like (R)‐ketamine, Lanicemine, Venlafaxine, Fluoxetine, and Agomelatine (AGO) has been closely linked to the composition of gut microbiota [49, 50]. More interestingly, researchers have found that drugs that regulate gut microbiota, such as antibiotics (Rifaximin, Isoalantolactone, and Minocycline) and proton pump inhibitors (Esomeprazole, etc.), can also influence psychological stress relief [51, 52, 53]. Traditional Chinese medicine formulas, herbal medicines, and their individual components (monomers or compounds) may also alleviate stress‐related disorders by impacting gut microbiota [54, 55].

### Addressing evaluation gaps in drug–microbiota interactions: exploring novel methodologies

**Figure 3:**
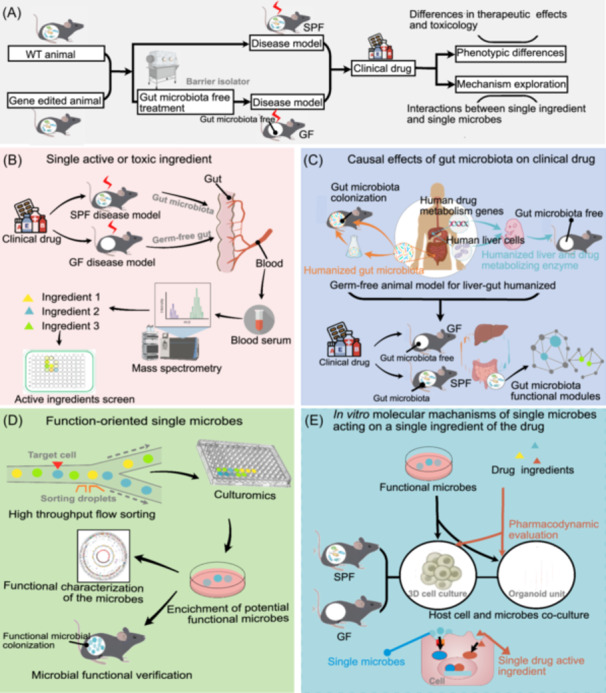
Drug preclinical research strategies based on precise germ‐free animal models. (A) Precise germ‐free animal models. Single functional microbe colonization, germ‐free treatment of genetically engineered disease animals, and liver‐gut humanization model system provide support for studying the in vivo function of gut microbiota in drug metabolism. (B) Gut microbiota in vivo function. The drug‐containing serum from GF disease model animal can not only screen small molecule pharmacodynamic substances modified by gut microbiota enzyme but also achieve more refined research of “host cell—gut microbiota—drug” by the intervention on host cells in vitro. (C) High‐resolution sequencing for single‐cell recognition. The single‐cell resolution sequencing of the microbiota (Microbe‐seq) can realize the high‐precision cognition on the cell individual level of the in vivo function of drug transformation. (D) Function‐oriented precise bacterial culture. The construction of GF animals with specific functional phenotypes will realize the transformation of small molecule drugs modified by microbial enzymes from in vitro function to in vivo function. (E) Coculture based on the in vivo function of gut microbiota. The microbiota‐loaded organoids are excellent media for host “cells and microbes” coculture.

The ability of human gut microbiota to metabolize small molecule medications contributes to variations in clinical drug efficacy. *Pharmacomicrobiomics* offers a promising avenue for preclinical studies to optimize drug effectiveness. However, current research faces limitations due to the complex interplay between gut microbiota and drugs in vivo. Traditionally, researchers have focused on isolating single bacterial strains from the gut to study drug metabolism mechanisms in vitro. While in vitro culture allows in‐depth analysis, it fails to capture the in vivo function of the microbiota—its interaction with host genes and its role in shaping the metabolic and immune environment within the body. To gain a complete picture, it is crucial to identify key gut microbiota‐driven pharmacodynamic ingredients within the unique in vivo environment of host cells and tissues, rather than relying solely on in vitro cultures. Next‐generation sequencing technologies have empowered researchers to understand the role of gut microbiota in drug metabolism in vivo. However, these techniques often lack the ability to establish causal relationships between specific microbes and drug metabolism. Sequencing data may reveal enzymes with similar functions across different bacterial species, making it difficult to pinpoint the functionally relevant bacteria. Furthermore, research has broadened its focus beyond the digestive and respiratory systems, recognizing the potential role of microbiota in other tissues in disease onset and progression [56, 57]. This raises the question of whether drug‐targeting microbiota resides not only in the gut but also at the site of drug delivery. Precise germ‐free animal models offer a potential solution to the challenges mentioned above (Figure 3A). These models, with their in vivo gut microbiota function, allow researchers to investigate the interaction between drugs and gut microbiota. Colonizing germ‐free animals with single, functionally characterized microbes can provide direct evidence for gut microbiota's involvement in drug metabolism in vivo, helping to elucidate the causal relationship between specific microbes and clinical drug efficacy and safety. Germ‐free treatment of genetically engineered animals can provide even deeper insights into the mechanisms of gut microbiota‐mediated drug metabolism. Additionally, addressing the challenge of “in vivo” gut microbiota function is crucial for understanding how gut microbiota influences environmental factors, leading to variations in drug effectiveness and IVDR. Furthermore, identifying microbes at the single‐cell level can provide technical support for isolating functionally relevant, yet difficult‐to‐cultivate, microbiota strains, given the low sequence conservation and functional redundancy within microbial genes. Ultimately, advancing *Pharmacomicrobiomics* research requires the development of in‐depth coculture models that integrate tissues, cells, and microbes, reflecting the “in vivo” function of gut microbiota.

### Leveraging in vivo gut microbiota function for the design of next‐generation drug ingredients involved in drug–microbiota interaction

Genetically engineered germ‐free disease models offer a top‐down approach to understanding the “in vivo” function of drug metabolism by gut microbiota (Figure 3B). These models can be used to screen for the active ingredients of bacterially modified drugs that enter the bloodstream [58]. This approach sheds light on how gut microbiota communities, functioning as a metabolic “factory,” modify pharmaceutical compounds through the action of microbial enzymes [59]. However, it is important to recognize that the metabolic effects of gut microbiota on drugs are also influenced by the host's unique immune and metabolic microenvironment. While drugs, whether oral or intravenous, are primarily metabolized in the liver before entering the intestine for absorption, the impact of gut microbiota on drug efficacy after this initial metabolism often goes overlooked. These modifications can occur through the action of microbial enzymes within the complex gut microbiota community. To effectively analyze the pharmacodynamic mechanisms of Traditional Chinese Medicine (TCM) formulas, TCM compounds, and other natural products with complex components, researchers may benefit from developing efficient techniques for genetic engineering and establishing aseptic/humanized microbiota animal models. This approach is particularly valuable for studying the interactions between gut microbiota, host, and drugs. Similar to the approach used in serum pharmacology for TCM and other complex natural compounds [60], researchers can utilize drug‐containing serum from GF disease model animals. This strategy allows not only for screening small molecule pharmacodynamic substances modified by gut microbiota enzymes but also for more refined in vitro studies investigating the interplay between host cell—gut microbiota—drug.

### High‐resolution sequencing for single‐cell recognition provides technology for new evaluation system for drug–microbial interaction research

Gut‐liver humanization models that incorporate human gut microbiota and drug‐metabolizing enzymes can offer a more clinically relevant platform for studying the interactions between gut microbiota, drugs, and humans (Figure 3C). High‐throughput sequencing plays a crucial role in characterizing gut microbiota by analyzing various aspects like the metagenome, transcriptome, metabolome, and isotope nucleic acid probe labeling coupled sequencing. However, most current sequencing methods decode all the genetic information within a sample, making it difficult to pinpoint the specific function of a single microbe in a complex gut microbiota community due to functional redundancy and low gene conservation among gut microbes. Microbe‐seq offers a powerful alternative—a high‐throughput technique that generates individual microbial genomes from complex communities. This allows researchers to investigate these communities at single‐cell resolution. Using Microbe‐seq, scientists have successfully obtained over 20,000 single‐amplified genomes (SAGs) from a single human donor [61, 62]. This unprecedented resolution allows for “in vivo” functional analysis of drug transformation at the individual cell level. Microbe‐seq holds promise for identifying gut microbiota that influence drug efficacy and toxicological effects in vivo. By eliminating the need for large‐scale microbial purification and culturing, this technique paves the way for function‐oriented, precise bacterial culture methods.

### Precise function‐oriented microbes culture enables a new evaluation system for drug–microbial interaction

Despite the remarkable progress achieved in gut microbiota research over the past few years, a significant portion of intestinal microbes remain unculturable. This presents a challenge for fully understanding the in vivo mechanisms of microbial drug metabolism and for large‐scale production of beneficial, microbially modified drug molecules. The human gut microbiome boasts a vast array of taxa, each carrying unique genes and gene families. However, a notable characteristic is the presence of phylogenetically diverse taxa that share similar genes and perform comparable functions [63]. This highlights the potential of “microbiota function‐oriented host‐microbiota crosstalk” as a new area of investigation. Microbe‐seq technology offers a powerful tool for single‐bacterium identification and sorting. This, combined with a strategy called *culturomics*, provides significant technical support for elucidating the role of gut microbiota in vivo function in clinical drug efficacy and safety (Figure 3D). Culturomics incorporates various growth conditions with rapid microbial identification techniques [64]. Furthermore, genetic manipulation techniques like CRISPR‐Cas9 enable editing of bacterial and fungal strains within the complex gut microbiome. This allows researchers to investigate the specific role of individual genes within this intricate system [63, 65]. These advancements highlight the growing sophistication of methods for mining functional bacteria. Functional genomics is another promising approach. Recent studies have demonstrated its potential for characterizing physiologically active biomolecules. For example, this approach can be applied to identify bacterial polysaccharide consumption loci targeting pectins, with expression levels corresponding to strain‐specific liberation [66]. Understanding the activity and substrate specificity of bacterial drug‐modifying enzymes is crucial, as these enzymes significantly impact the activity of drug components. Existing research has successfully characterized the activity and substrate specificity of bacterial enzymes from culturable bacteria, particularly in the context of inflammatory diseases [67]. Finally, the construction of germ‐free animals with specific functional phenotypes offers a promising avenue for translating the “in vitro” function of microbially modified small molecule drugs to their “in vivo” function.

### Coculture, models mimicking gut microbiota function: A novel platform for drug–microbiota interaction evaluation

Three‐dimensional in vitro cell culture systems known as “organoids” offer a powerful tool for studying drug effects. These models closely resemble the source tissues or organs in vivo by replicating their complex spatial morphology and differentiated cell types [68]. Microbiota‐loaded organoids provide an excellent platform for coculturing host cells and microbes, allowing researchers to investigate drug pharmacology within the context of “microbiota–host” interactions (Figure 3E). The brain, bone, and blood have long been considered sterile environments. However, with the advent of high‐resolution sequencing techniques, growing evidence suggests that microbiota, including bacteria, fungi, and viruses, colonize various organs and play a crucial physiological role within the host's local tissue microenvironment [57, 69]. Particularly, noteworthy is the presence of microbiota in tumor tissues, cells, and blood, where they appear to influence host immunity and cancer cell metastasis [69, 70]. An intriguing question remains unanswered: will drugs delivered to specific host tissues or cells undergo secondary metabolism and modification by local microbiota, potentially impacting their efficacy? While current research has not yet focused on the role and mechanisms of local microbiota in influencing the effectiveness and toxicity of drugs targeted for localized delivery, these are undoubtedly issues that will capture the attention of researchers in the near future.

This study addresses two key issues regarding clinical drugs and gut microbiota. First, individual variability in the human body arises from a combination of genetic and environmental factors. A critical question remains: how do these variations in the human microenvironment, including gut microbiota, affect the safety and efficacy of medications? Second, *pharmacogenomics* sheds light on how individual genetic variations influence drug absorption, distribution, metabolism, and excretion. However, research on the impact of environmental factors, particularly gut microbiota, on drug ADME remains limited. Given the gut microbiota's crucial role in gene–environment interactions affecting drug ADME, it presents a valuable and understudied avenue for preclinical drug safety and efficacy investigations. Our proposed new evaluation system for drug‐microbiota interaction shows significant promise, as evidenced by the findings above. Future research can explore several exciting avenues. First, by comparing conventional and germ‐free animals, disease models with humanized gut microbiota, and single‐strain animal drug metabolism models, researchers can develop preclinical evaluation technology services. This approach can also optimize drug safety and efficacy testing, identify potential microbial targets for existing drugs, and aid in discovering new drug mechanisms and uses. Second, research could focus on evaluating the effectiveness of clinical interventions targeting gut microbiota in vivo, such as vaccines, antibodies, and cell‐based therapies. Finally, the development of a comprehensive technical service platform integrating animal models, microbiome technology, and differential drug response analysis would be a valuable resource for studying drug‐microbiota interactions. In conclusion, the strong operability and high safety of gut microbial regulation in germ‐free animal models make them a promising new tool for evaluating drug–microbiota interactions.

## AUTHOR CONTRIBUTIONS

Tian‐Hao Liu, Chen‐Yang Zhang, Hang Zhang, Xiu‐Wu Bian, Wei Hong, Yu‐Zheng Xue participated in study design. Tian‐Hao Liu, Xue Li, Ya‐Hong Zhou, Jing Jin, Shi‐Qiang Liang conducted study operation. Chen‐Yang Zhang, Tian‐Hao Liu, Yu‐Zheng Xue, Feng‐Lai Yuan helped to draft and revise the manuscript. All authors have read the final manuscript and approved it for publication.

## CONFLICT OF INTEREST STATEMENT

The authors declare no conflict of interest.

## ETHICS STATEMENT

No animals or humans were involved in this study.

## Data Availability

No new data was generated in this study. Supplementary materials (graphical abstract, slides, videos, Chinese translated version and update materials) may be found in the online DOI or iMeta Science (http://www.imeta.science/).

## References

[1] Zhao, Qing , Yao Chen , Weihua Huang , Honghao Zhou , and Wei Zhang . 2023. “Drug‐Microbiota Interactions: An Emerging Priority for Precision Medicine.” Signal Transduction and Targeted Therapy 8: 386. 10.1038/s41392-023-01619-w 37806986 PMC10560686

[2] Harrer, Stefan , Pratik Shah , Bhavna Antony , and Jianying Hu . 2019. “Artificial Intelligence for Clinical Trial Design.” Trends in Pharmacological Sciences 40: 577–591. 10.1016/j.tips.2019.05.005 31326235

[3] Rothschild, Daphna , Omer Weissbrod , Elad Barkan , Alexander Kurilshikov , Tal Korem , David Zeevi , Paul I. Costea , et al. 2018. “Environment Dominates Over Host Genetics in Shaping Human Gut Microbiota.” Nature 555: 210–215. 10.1038/nature25973 29489753

[4] Gacesa, R. , A. Kurilshikov , A. Vich Vila , T. Sinha , M. A. Y. Klaassen , L. A., Bolte , S. Andreu‐Sánchez , et al. 2022. “Environmental Factors Shaping the Gut Microbiome in a Dutch Population.” Nature 604: 732–739. 10.1038/s41586-022-04567-7 35418674

[5] Wang, Kai , Zhiwei Zhang , Jing Hang , Jia Liu , Fusheng Guo , Yong Ding , Meng Li , et al. 2023. “Microbial‐Host‐Isozyme Analyses Reveal Microbial DPP4 as a Potential Antidiabetic Target.” Science 381: eadd5787. 10.1126/science.add5787 37535747

[6] Pant, Archana , Tushar K. Maiti , Dinesh Mahajan , and Bhabatosh Das . 2023. “Human Gut Microbiota and Drug Metabolism.” Microbial Ecology 86: 97–111. 10.1007/s00248-022-02081-x 35869999 PMC9308113

[7] Liu, Na , Jinfeng Liu , Binjie Zheng , Xiangchang Zeng , Zixin Ye , Xinyi Huang , Wenhui Liu , et al. 2023. “Gut Microbiota Affects Sensitivity to Immune‐Mediated Isoniazid‐Induced Liver Injury.” Biomedicine & Pharmacotherapy 160: 114400. 10.1016/j.biopha.2023.114400 36805186

[8] Malesza, Ida Judyta , Michał Malesza , Jarosław Walkowiak , Nadiar Mussin , Dariusz Walkowiak , Raisa Aringazina , Joanna Bartkowiak‐Wieczorek , and Edyta Mądry . 2021. “High‐Fat, Western‐Style Diet, Systemic Inflammation, and Gut Microbiota: A Narrative Review.” Cells 10: 3164. 10.3390/cells10113164 34831387 PMC8619527

[9] Nagata, Naoyoshi , Suguru Nishijima , Tohru Miyoshi‐Akiyama , Yasushi Kojima , Moto Kimura , Ryo Aoki , Mitsuru Ohsugi , et al. 2022. “Population‐Level Metagenomics Uncovers Distinct Effects of Multiple Medications on the Human Gut Microbiome.” Gastroenterology 163: 1038–1052. 10.1053/j.gastro.2022.06.070 35788347

[10] Cai, Jie , Lulu Sun , and Frank J. Gonzalez . 2022. “Gut Microbiota‐Derived Bile Acids in Intestinal Immunity, Inflammation, and Tumorigenesis.” Cell Host & Microbe 30: 289–300. 10.1016/j.chom.2022.02.004 35271802 PMC8923532

[11] Winston, Jenessa A. , and Casey M. Theriot . 2020. “Diversification of Host Bile Acids by Members of the Gut Microbiota.” Gut Microbes 11: 158–171. 10.1080/19490976.2019.1674124 31595814 PMC7053883

[12] Zimmermann, Michael , Maria Zimmermann‐Kogadeeva , Rebekka Wegmann , and Andrew L. Goodman . 2019. “Mapping Human Microbiome Drug Metabolism by Gut Bacteria and Their Genes.” Nature 570: 462–467. 10.1038/s41586-019-1291-3 31158845 PMC6597290

[13] Haiser, Henry J. , David B. Gootenberg , Kelly Chatman , Gopal Sirasani , Emily P. Balskus , and Peter J. Turnbaugh . 2013. “Predicting and Manipulating Cardiac Drug Inactivation by the Human Gut Bacterium Eggerthella Lenta.” Science 341: 295–298. 10.1126/science.1235872 23869020 PMC3736355

[14] Clayton, T. Andrew , David Baker , John C. Lindon , Jeremy R. Everett , and Jeremy K. Nicholson . 2009. “Pharmacometabonomic Identification of a Significant Host‐Microbiome Metabolic Interaction Affecting Human Drug Metabolism.” Proceedings of National Acadamy of Sciences of the United States of America 106: 14728–14733. 10.1073/pnas.0904489106 PMC2731842 19667173

[15] Wallace, Bret D. , Hongwei Wang , Kimberly T. Lane , John E. Scott , Jillian Orans , Ja Seol Koo , Madhukumar Venkatesh , et al. 2010. “Alleviating Cancer Drug Toxicity by Inhibiting a Bacterial Enzyme.” Science 330: 831–835. 10.1126/science.1191175 21051639 PMC3110694

[16] van Kessel, Sebastiaan P. , Alexandra K. Frye , Ahmed O. El‐Gendy , Maria Castejon , Ali Keshavarzian , Gertjan van Dijk , Sahar El Aidy , et al. 2019. “Gut Bacterial Tyrosine Decarboxylases Restrict Levels of Levodopa in the Treatment of Parkinson's Disease.” Nature Communications 10: 310. 10.1038/s41467-019-08294-y PMC6338741 30659181

[17] Vourakis, Margaret , Gaétan Mayer , and Guy Rousseau . 2021. “The Role of Gut Microbiota on Cholesterol Metabolism in Atherosclerosis.” International Journal of Molecular Sciences 22: 8074. 10.3390/ijms22158074 34360839 PMC8347163

[18] Chiocchetti, G. M. , D. Vélez , and V. Devesa . 2018. “Effect of Subchronic Exposure to Inorganic Arsenic on the Structure and Function of the Intestinal Epithelium.” Toxicology Letters 286: 80–88. 10.1016/j.toxlet.2018.01.011 29355690

[19] Hojo, Mariko , Takashi Asahara , Akihito Nagahara , Tsutomu Takeda , Kohei Matsumoto , Hiroya Ueyama , Kenshi Matsumoto , et al. 2018. “Gut Microbiota Composition Before and After Use of Proton Pump Inhibitors.” Digestive Diseases and Science 63: 2940–2949. 10.1007/s10620-018-5122-4 PMC6182435 29796911

[20] Zhou, Bailing , Xuyang Xia , Peiqi Wang , Shuang Chen , Chaoheng Yu , Rong Huang , Rui Zhang , et al. 2018. “Induction and Amelioration of Methotrexate‐Induced Gastrointestinal Toxicity are Related to Immune Response and Gut Microbiota.” Ebiomedicine 33: 122–133. 10.1016/j.ebiom.2018.06.029 30049384 PMC6085585

[21] Vallianou, Natalia G. , Theodora Stratigou , and Stylianos Tsagarakis . 2019. “Metformin and Gut Microbiota: Their Interactions and Their Impact on Diabetes.” Hormones 18: 141–144. 10.1007/s42000-019-00093-w 30719628

[22] Anthony, Winston E. , Carey‐Ann D. Burnham , Gautam Dantas , and Jennie H. Kwon . 2021. “The Gut Microbiome as a Reservoir for Antimicrobial Resistance.” The Journal of Infectious Diseases 223: S209–S213. 10.1093/infdis/jiaa497 33326581 PMC8206794

[23] Thänert, Robert , Sanjam S. Sawhney , Drew J. Schwartz , and Gautam Dantas . 2022. “The Resistance Within: Antibiotic Disruption of the Gut Microbiome and Resistome Dynamics in Infancy.” Cell Host & Microbe 30: 675–683. 10.1016/j.chom.2022.03.013 35550670 PMC9173668

[24] Wang, Yi‐Fei , Min Qiao , Dong Zhu , and Yong‐Guan Zhu . 2020. “Antibiotic Resistance in the Collembolan Gut Microbiome Accelerated by the Nonantibiotic Drug Carbamazepine.” Environmental Science & Technology 54: 10754–10762. 10.1021/acs.est.0c03075 32816468

[25] Bakkeren, Erik , Jana S. Huisman , Stefan A. Fattinger , Annika Hausmann , Markus Furter , Adrian Egli , Emma Slack , et al. 2019. “Salmonella Persisters Promote the Spread of Antibiotic Resistance Plasmids in the Gut.” Nature 573: 276–280. 10.1038/s41586-019-1521-8 31485077 PMC6744281

[26] Hoyos, Pilar , Almudena Perona , Teodora Bavaro , Francesca Berini , Flavia Marinelli , Marco Terreni , and María J. Hernáiz . 2022. “Biocatalyzed Synthesis of Glycostructures With Anti‐Infective Activity.” Accounts of Chemical Research 55: 2409–2424. 10.1021/acs.accounts.2c00136 35942874 PMC9454102

[27] Park, Joon Seok , Francesca S. Gazzaniga , Meng Wu , Amalia K. Luthens , Jacob Gillis , Wen Zheng , Martin W. LaFleur , et al. 2023. “Publisher Correction: Targeting PD‐L2‐RGMb Overcomes Microbiome‐Related Immunotherapy Resistance.” Nature 618: E27. 10.1038/s41586-023-06237-8 37264079 PMC12025598

[28] Vieira‐Silva, Sara , Gwen Falony , Eugeni Belda , Trine Nielsen , Judith Aron‐Wisnewsky , Rima Chakaroun , Sofia K. Forslund , et al. 2020. “Statin Therapy is Associated With Lower Prevalence of Gut Microbiota Dysbiosis.” Nature 581: 310–315. 10.1038/s41586-020-2269-x 32433607

[29] Roberti, Maria Paula , Satoru Yonekura , Connie P. M. Duong , Marion Picard , Gladys Ferrere , Maryam Tidjani Alou , Conrad Rauber , et al. 2020. “Chemotherapy‐Induced Ileal Crypt Apoptosis and the Ileal Microbiome Shape Immunosurveillance and Prognosis of Proximal Colon Cancer.” Nature Medicine 26: 919–931. 10.1038/s41591-020-0882-8 32451498

[30] Teng, Teng , Guodong Sun , Hongwei Ding , Xin Song , Guangdong Bai , Baoming Shi , and Tingting Shang . 2023. “Characteristics of Glucose and Lipid Metabolism and the Interaction Between Gut Microbiota and Colonic Mucosal Immunity in Pigs During Cold Exposure.” Journal of Animal Science and Biotechnology 14: 84. 10.1186/s40104-023-00886-5 37400906 PMC10318708

[31] Nagai, Minami , Miyu Moriyama , Chiharu Ishii , Hirotake Mori , Hikaru Watanabe , Taku Nakahara , Takuji Yamada , et al. 2023. “High Body Temperature Increases Gut Microbiota‐Dependent Host Resistance to Influenza A Virus and SARS‐CoV‐2 Infection.” Nature Communication 14: 3863. 10.1038/s41467-023-39569-0 PMC10313692 37391427

[32] Zhao, Anpeng , Wenbin Li , and Rong Wang . 2023. “The Influences and Mechanisms of High‐Altitude Hypoxia Exposure on Drug Metabolism.” Current Drug Metabolism 24: 152–161. 10.2174/1389200224666221228115526 36579391

[33] Zhao, Chen , Chenyu Li , Xiaoming Wang , Zhuosong Cao , Chao Gao , Sicong Su , Bin Xue , et al. 2022. “Monitoring and Evaluation of Antibiotic Resistance Genes in Three Rivers in Northeast China.” Environmental Science and Pollution Research 29: 44148–44161. 10.1007/s11356-022-18555-x 35122641

[34] Li, Shengnan , Chaofan Zhang , Fengxiang Li , Tao Hua , Qixing Zhou , and Shih‐Hsin Ho . 2021. “Technologies Towards Antibiotic Resistance Genes (ARGs) Removal From Aquatic Environment: A Critical Review.” Journal of Hazardous Materials 411: 125148. 10.1016/j.jhazmat.2021.125148 33486226

[35] Maurya, Anand Prakash , Jina Rajkumari , and Piyush Pandey . 2021. “Enrichment of Antibiotic Resistance Genes (ARGs) in Polyaromatic Hydrocarbon‐Contaminated Soils: A Major Challenge for Environmental Health.” Environmental Science and Pollution Research 28: 12178–12189. 10.1007/s11356-020-12171-3 33394421

[36] Liu, Jun‐Xi , Hong‐Yu Yuan , Ya‐Nan Li , Zhen Wei , Yang Liu , and Jun Liang . 2022. “ *Ephedra sinica* Polysaccharide Alleviates Airway Inflammations of Mouse Asthma‐Like Induced by Pm2.5 and Ovalbumin via the Regulation of Gut Microbiota and Short Chain Fatty Acid.” Journal of Pharmacy and Pharmacology 74: 1784–1796. 10.1093/jpp/rgac078 36301619

[37] Shi, Chunzhen , Xiaohong Han , Wei Guo , Qi Wu , Xiaoxi Yang , Yuanyuan Wang , Gang Tang , et al. 2022. “Disturbed Gut‐Liver Axis Indicating Oral Exposure to Polystyrene Microplastic Potentially Increases the Risk of Insulin Resistance.” Environment International 164: 107273. 10.1016/j.envint.2022.107273 35526298

[38] Chen, Xuebing , Jingshen Zhuang , Qianling Chen , Luyao Xu , Xia Yue , and Dongfang Qiao . 2022. “Chronic Exposure to Polyvinyl Chloride Microplastics Induces Liver Injury and Gut Microbiota Dysbiosis Based on the Integration of Liver Transcriptome Profiles and Full‐Length 16S rRNA Sequencing Data.” Science of The Total Environment 839: 155984. 10.1016/j.scitotenv.2022.155984 35588832

[39] Jing, Jiaru , Lei Zhang , Lin Han , Jingyu Wang , Wei Zhang , Ziyan Liu , and Ai Gao . 2022. “Polystyrene Micro‐/Nanoplastics Induced Hematopoietic Damages via the Crosstalk of Gut Microbiota, Metabolites, and Cytokines.” Environment International 161: 107131. 10.1016/j.envint.2022.107131 35149446

[40] Lin, Hui , Liping Meng , Zhenzhu Sun , Shiming Sun , Xingxiao Huang , Na Lin , Jie Zhang , et al. 2021. “Yellow Wine Polyphenolic Compound Protects Against Doxorubicin‐Induced Cardiotoxicity by Modulating the Composition and Metabolic Function of the Gut Microbiota.” Circulation: Heart Failure 14: e8220. 10.1161/CIRCHEARTFAILURE.120.008220 34665676

[41] Wang, Meifang , Yinping Dong , Jing Wu , Hongyan Li , Yuanyang Zhang , Saijun Fan , and Deguan Li . 2020. “Baicalein Ameliorates Ionizing Radiation‐Induced Injuries by Rebalancing Gut Microbiota and Inhibiting Apoptosis.” Life Sciences 261: 118463. 10.1016/j.lfs.2020.118463 32950576

[42] Hu, Jinglu , Wencheng Jiao , Ziyan Tang , Chunqing Wang , Qi Li , Meng Wei , Shiyong Song , et al. 2023. “Quercetin Inclusion Complex Gels Ameliorate Radiation‐Induced Brain Injury by Regulating Gut Microbiota.” Biomedicine & Pharmacotherapy 158: 114142. 10.1016/j.biopha.2022.114142 36527844

[43] Zheng, Ying , Xu Pang , Xiaoxia Zhu , Zhiyun Meng , Xiaojuan Chen , Jie Zhang , Qianzhi Ding , et al. 2021. “ *Lycium barbarum* Mitigates Radiation Injury via Regulation of the Immune Function, Gut Microbiota, and Related Metabolites.” Biomedicine & Pharmacotherapy 139: 111654. 10.1016/j.biopha.2021.111654 33957563

[44] Xu, Chunliang , Sung Kyun Lee , Dachuan Zhang , and Paul S. Frenette . 2020. “The Gut Microbiome Regulates Psychological‐Stress‐Induced Inflammation.” Immunity 53: 417–428. 10.1016/j.immuni.2020.06.025 32735844 PMC7461158

[45] Wu, Wei‐Li , Mark D. Adame , Chia‐Wei Liou , Jacob T. Barlow , Tzu‐Ting Lai , Gil Sharon , Catherine E Schretter , et al. 2021. “Microbiota Regulate Social Behaviour via Stress Response Neurons in the Brain.” Nature 595: 409–414. 10.1038/s41586-021-03669-y 34194038 PMC8346519

[46] Zemanová, Nina , Pavel Anzenbacher , Iveta Zapletalová , Lenka Jourová , Petra Hermanová , Tomáš Hudcovic , Hana Kozáková , et al. 2020. “The Role of the Microbiome and Psychosocial Stress in the Expression and Activity of Drug Metabolizing Enzymes in Mice.” Science Reports 10: 8529. 10.1038/s41598-020-65595-9 PMC7244717 32444678

[47] Ma, Junxing , Ran Wang , Yaoxing Chen , Zixu Wang , and Yulan Dong . 2023. “5‐HT Attenuates Chronic Stress‐Induced Cognitive Impairment in Mice Through Intestinal Flora Disruption.” Journal of Neuroinflammation 20: 23. 10.1186/s12974-023-02693-1 36737776 PMC9896737

[48] Mir, Hayatte‐Dounia , Alexandre Milman , Magali Monnoye , Véronique Douard , Catherine Philippe , Agnès Aubert , Nathalie Castanon , et al. 2020. “The Gut Microbiota Metabolite Indole Increases Emotional Responses and Adrenal Medulla Activity in Chronically Stressed Male Mice.” Psychoneuroendocrinology 119: 104750. 10.1016/j.psyneuen.2020.104750 32569990

[49] An, Qi , Chungen Li , Yaxing Chen , Yang Yang , Rao Song , LiangXue Zhou , Jiong Li , et al. 2020. “Scaffold Hopping of Agomelatine Leads to Enhanced Antidepressant Effects by Modulation of Gut Microbiota and Host Immune Responses.” Pharmacology Biochemistry and Behavior 192: 172910. 10.1016/j.pbb.2020.172910 32194087

[50] Yang, Chun , Youge Qu , Yuko Fujita , Qian Ren , Min Ma , Chao Dong , and Kenji Hashimoto . 2017. “Possible Role of the Gut Microbiota‐Brain Axis in the Antidepressant Effects of (R)‐Ketamine in a Social Defeat Stress Model.” Translational Psychiatry 7: 1294. 10.1038/s41398-017-0031-4 29249803 PMC5802627

[51] Li, Haonan , Yujiao Xiang , Zemeng Zhu , Wei Wang , Zhijun Jiang , Mingyue Zhao , Shuyue Cheng , et al. 2021. “Rifaximin‐Mediated Gut Microbiota Regulation Modulates the Function of Microglia and Protects Against CUMS‐Induced Depression‐Like Behaviors in Adolescent Rat.” Journal of Neuroinflammation 18: 254. 10.1186/s12974-021-02303-y 34736493 PMC8567657

[52] Wang, Siming , Youge Qu , Lijia Chang , Yaoyu Pu , Kai Zhang , and Kenji Hashimoto . 2020. “Antibiotic‐Induced Microbiome Depletion is Associated With Resilience in Mice After Chronic Social Defeat Stress.” Journal of Affective Disorders 260: 448–457. 10.1016/j.jad.2019.09.064 31539679

[53] Meng, Chen , Siyuan Feng , Zikai Hao , Chen Dong , and Hong Liu . 2022. “Antibiotics Exposure Attenuates Chronic Unpredictable Mild Stress‐Induced Anxiety‐Like and Depression‐Like Behavior.” Psychoneuroendocrinology 136: 105620. 10.1016/j.psyneuen.2021.105620 34896741

[54] Ge, Ping‐Yuan , Shu‐Yue Qu , Sai‐jia Ni , Zeng‐Ying Yao , Yi‐Yu Qi , Xin Zhao , Rui Guo , et al. 2023. “Berberine Ameliorates Depression‐Like Behavior in CUMS Mice by Activating TPH1 and Inhibiting IDO1‐Associated With Tryptophan Metabolism.” Phytotherapy Research 37: 342–357. 10.1002/ptr.7616 36089660

[55] Zhao, Feng , Suzhen Guan , Youjuan Fu , Kai Wang , Zhihong Liu , and Tzi Bun Ng . 2021. “ *Lycium barbarum* Polysaccharide Attenuates Emotional Injury of Offspring Elicited by Prenatal Chronic Stress in Rats via Regulation of Gut Microbiota.” Biomedicine & Pharmacotherapy 143: 112087. 10.1016/j.biopha.2021.112087 34474339

[56] Nejman, Deborah , Ilana Livyatan , Garold Fuks , Nancy Gavert , Yaara Zwang , Leore T. Geller , Aviva Rotter‐Maskowitz , et al. 2020. “The Human Tumor Microbiome is Composed of Tumor Type‐Specific Intracellular Bacteria.” Science 368: 973–980. 10.1126/science.aay9189 32467386 PMC7757858

[57] Fu, Aikun , Bingqing Yao , Tingting Dong , Yongyi Chen , Jia Yao , Yu Liu , Hang Li , et al. 2022. “Tumor‐Resident Intracellular Microbiota Promotes Metastatic Colonization in Breast Cancer.” Cell 185: 1356–1372. 10.1016/j.cell.2022.02.027 35395179

[58] Nemet, Ina , Prasenjit Prasad Saha , Nilaksh Gupta , Weifei Zhu , Kymberleigh A. Romano , Sarah M. Skye , Tomas Cajka , et al. 2020. “A Cardiovascular Disease‐Linked Gut Microbial Metabolite Acts via Adrenergic Receptors.” Cell 180: 862–877. 10.1016/j.cell.2020.02.016 32142679 PMC7402401

[59] Ren, Lujing , Cheng Peng , Xuechao Hu , Yiwen Han , and He Huang . 2020. “Microbial Production of Vitamin K2: Current Status and Future Prospects.” Biotechnology Advances 39: 107453. 10.1016/j.biotechadv.2019.107453 31629792

[60] Han, Ying , Hui Sun , Aihua Zhang , Guangli Yan , and Xi‐Jun Wang . 2020. “Chinmedomics, a New Strategy for Evaluating the Therapeutic Efficacy of Herbal Medicines.” Pharmacology & Therapeutics 216: 107680. 10.1016/j.pharmthera.2020.107680 32956722 PMC7500400

[61] Zheng, Wenshan , Shijie Zhao , Yehang Yin , Huidan Zhang , David M. Needham , Ethan D. Evans , Chengzhen L. Dai , et al. 2022. “High‐Throughput, Single‐Microbe Genomics With Strain Resolution, Applied to a Human Gut Microbiome.” Science 376: m1483. 10.1126/science.abm1483 35653470

[62] Lloréns‐Rico, Verónica , Joshua A. Simcock , Geert R. B. Huys , and Jeroen Raes . 2022. “Single‐Cell Approaches in Human Microbiome Research.” Cell 185: 2725–2738. 10.1016/j.cell.2022.06.040 35868276

[63] Tian, Liang , Xu‐Wen Wang , Ang‐Kun Wu , Yuhang Fan , Jonathan Friedman , Amber Dahlin , Matthew K Waldor , et al. 2020. “Deciphering Functional Redundancy in the Human Microbiome.” Nature Communication 11: 6217. 10.1038/s41467-020-19940-1 PMC7719190 33277504

[64] Lagier, Jean‐Christophe , Grégory Dubourg , Matthieu Million , Frédéric Cadoret , Melhem Bilen , Florence Fenollar , Anthony Levasseur , et al. 2018. “Culturing the Human Microbiota and Culturomics.” Nature Reviews Microbiology 16: 540–550. 10.1038/s41579-018-0041-0 29937540

[65] Li, Xin V. , Irina Leonardi , Gregory G. Putzel , Alexa Semon , William D. Fiers , Takato Kusakabe , Woan‐Yu Lin , et al. 2022. “Immune Regulation by Fungal Strain Diversity in Inflammatory Bowel Disease.” Nature 603: 672–678. 10.1038/s41586-022-04502-w 35296857 PMC9166917

[66] Han, Nathan D. , Jiye Cheng , Omar Delannoy‐Bruno , Daniel Webber , Nicolas Terrapon , Bernard Henrissat , Dmitry A. Rodionov , et al. 2022. “Microbial Liberation of N‐Methylserotonin From Orange Fiber in Gnotobiotic Mice and Humans.” Cell 185: 2495–2509. 10.1016/j.cell.2022.06.004 35764090 PMC9271604

[67] Luis, Ana S. , Chunsheng Jin , Gabriel Vasconcelos Pereira , Robert W. P. Glowacki , Sadie R. Gugel , Shaleni Singh , Dominic P. Byrne , et al. 2021. “A Single Sulfatase is Required to Access Colonic Mucin by a Gut Bacterium.” Nature 598: 332–337. 10.1038/s41586-021-03967-5 34616040 PMC9128668

[68] Nejman, Deborah , Ilana Livyatan , Garold Fuks , Nancy Gavert , Yaara Zwang , Leore T. Geller , Aviva Rotter‐Maskowitz , et al. 2020. “The Human Tumor Microbiome is Composed of Tumor Type‐Specific Intracellular Bacteria.” Science 368: 973–980. 10.1126/science.aay9189 32467386 PMC7757858

[69] Narunsky‐Haziza, Lian , Gregory D. Sepich‐Poore , Ilana Livyatan , Omer Asraf , Cameron Martino , Deborah Nejman , Nancy Gavert , et al. 2022. “Pan‐Cancer Analyses Reveal Cancer‐Type‐Specific Fungal Ecologies and Bacteriome Interactions.” Cell 185: 3789–3806. 10.1016/j.cell.2022.09.005 36179670 PMC9567272

[70] Harimoto, Tetsuhiro , Dhruba Deb , and Tal Danino . 2022. “A Rapid Screening Platform to Coculture Bacteria Within Tumor Spheroids.” Nature Protocols 17: 2216–2239. 10.1038/s41596-022-00723-5 35906291 PMC10332800

